# Androgen metabolism and inhibition of interleukin-1 synthesis in primary cultured human synovial macrophages

**DOI:** 10.1155/S096293519500024X

**Published:** 1995-03

**Authors:** M. Cutolo, S. Accardo, B. Villaggio, A. Barone, A. Sulli, E. Balleari, C. Bason, L. Felli, O. M. Granata, R. Amodio, L. Castagnetta

**Affiliations:** 1 Division of Rheumatology University of Genova Viale Benedetto XV Genova 16132 Italy; 2 Department of Internal Medicine University of Genova Viale Benedetto XV Genova 16132 Italy; 3 Division of Nephrology University of Genova Viale Benedetto XV Genova 16132 Italy; 4 Division of Orthopedic Surgery University of Genova Viale Benedetto XV Genova 16132 Italy; 5 Hormone Biochemistry Laboratories University of Palermo Italy

## Abstract

The presence of androgen receptors on synovial macrophages in human normal and rheumatoid synovial tissues has been described previously. It is now reported that primary cultured human macrophages obtained from normal and rheumatoid synovia express functional androgen receptors. We have investigated the capacity of cultured macrophages to metabolize androgens and have found that these cells were capable of metabolizing testosterone to the bioactive metabolite dihydrotestosterone. Therefore, macrophages contain the key enzymes of steroidogenesis, in particular the 5α-treductase. Furthermore, interleukin-1β production by primary cultured rheumatoid macrophages was analysed, following exposure to physiological concentrations of testosterone (10^−8^ M). A significant decrease of IL-1β levels in conditioned media after 24 h (p < 0.05) was observed. It is concluded that androgens may act directly on human macrophages and may interfere with some of their functions via receptor-dependent mechanisms.


Research Paper

Mediators of Inflammation 4, 138–143 (1995)

### Androgen metabolism and inhibition of interleukin-1 synthesis in primary cultured human synovial macrophages

M. Cutolo,[1,CA] S. Accardo,[1] B. Villaggio,[3] A. Barone,[1] A. Sulli,[1] E. Balleari,[2] C. Bason,[2] L. Felli,[4] O. M. Granata,[5] R. Amodio[4] and L. Castagnetta[5]

[1]Division of Rheumatology, [2]Department of Internal Medicine, [3]Division of Nephrology, and [4]Division of Orthopedic Surgery, University of Genova, Viale Benedetto XV, Genova, Italy 16132; and [5]Hormone Biochemistry Laboratories, University of Palermo, Italy

[CA] Corresponding Author

**THE presence of androgen receptors on synovial macrophages in human normal and rheumatoid synovial tissues has been described previously. It is now reported that primary cultured human macrophages obtained from normal and rheumatoid synovia express functional androgen receptors. We have investigated the capacity of cultured macrophages to metabolize androgens and have found that these cells were capable of metabolizing testosterone to the bioactive metabolite dihydrotestosterone. Therefore, macrophages contain the key enzymes of steroidogenesis, in particular the 5α-reductase. Furthermore, interleukin-1β production by primary cultured rheumatoid macrophages was analysed, following exposure to physiological concentrations of testosterone ($10^{-8}$ M). A significant decrease of IL-1β levels in conditioned media after 24 h ($p < 0.05$) was observed. It is concluded that androgens may act directly on human macrophages and may interfere with some of their functions via receptor-dependent mechanisms.**

**Key words:** Androgens, Cytokines, Interleukin-1, Receptors, Rheumatoid arthritis, Sex hormones, Synovial tissue

### Introduction

A sex-related dimorphism in immune capabilities is thought to be related to the physiological effects of sex hormones on the immune system.[1–5] Generally, at least at physiological concentrations, oestrogens exert immunostimulatory effects, whereas androgens are immunosuppressive and a preponderance of autoimmune diseases in females compared with males is well recognized.[6] In rheumatoid arthritis (RA), the synovial tissue is widely infiltrated by mononuclear cells and is considered the target tissue for a 'sexual dimorphism' in the immune response.[2–7] Sex hormones seem to play a role in immunomodulation via receptor-mediated mechanisms.

High affinity androgen receptors (AR) have already been detected in human maturing thymocytes.[8–11] However, it is only recently that androgen and oestrogen receptors have been detected in synovial macrophages (mϕs) of normal and rheumatoid synovial tissues, by immunostaining of cryosections and by biochemical characterization in homogenized tissues.[12,13] The presence of androgen receptors on mϕs induced us to analyse the metabolic pathway for androgens.

Mϕs were found to be capable of effectively metabolizing testosterone (Tes) to the bioactive dihydrotestosterone (DHT). In addition, since interleukin-1β (IL-1β) is mainly synthesized by activated macrophages and mediates the T- and B-lymphocyte stimulation during the immune response, we investigated the influence of androgens on IL-1β production by using cultured RA mϕs. It was found that physiological concentrations of testosterone ($10^{-8}$ M) significantly decreased IL-1β content in conditioned media.

In conclusion we proved that human mϕs express functional androgen receptors and should be modulated at least in some of their immune-related functions (i.e. IL-1β production) by the sex hormones that they actively metabolize.

### Materials and Methods

*Reagents:* Anti-androgen receptor mAbs that are known to react to human androgen and that do not cross-react with other receptors or with glucocorticoid receptors, were purchased from BioReagents (Neshanic Station, NJ, USA). Anti-macrophage associated antigens, Ber-MAC3 and MAC387 mAbs were obtained from Dakopatts (Copenhagen, Denmark). Tritiated testosterone and tritiated mibolerone were obtained from Amersham (Buckinghamshire, UK). IL-1β solid phase enzyme immunoassay (EIA) was obtained from Medgenix Diagnostics (Fleurus, Belgium). Biotin–streptavidin-amplified detection system was purchased from BioGenex Laboratories (San Ramon, CA, USA) and collagenase was from Sigma Chemical Co. (St Louis, MO, USA).

© 1995 Rapid Communications of Oxford Ltd

*Preparation of synovial tissue samples and synoviocyte culture:* Synovial tissue samples were obtained from six women (mean age 49.6 ± 14 years) and six men (mean age 53.7 ± 15 years) who fulfilled the American College of Rheumatology criteria for adult RA and were undergoing arthroscopic synovectomy of the knee.[14] Synovial samples from age and sex-matched subjects undergoing diagnostic arthroscopy for recent trauma of the knee (<10 days) were used as controls.[15] All the RA patients were found to be affected by Steinbrocker class II RA. At the time of surgery all the patients were being treated exclusively with nonsteroidal anti-inflammatory drugs. None had received any oral or intraarticular corticosteroid therapy and slow-acting antirheumatic drugs during the 4 months preceding the investigation, nor was the use of the oral contraceptive pill, 1,25-dihydroxyvitamin D3 or any hormone replacement therapy referred by the patients.[16–18] All subjects had normal liver, renal, prostate and thyroid functions and were within 20% of ideal body weight.

Freshly obtained tissue samples from the synovial lining were placed on ice, and stored at –20°C in 30% sucrose–50% glycerol buffer (v/v) (0.25 M sucrose, 10 mM HEPES and 1.5 mM $MgCl_2$, pH 7.4) until analysis for sex hormone receptors. Under these conditions, the concentration and molecular form of the receptor have been shown to be stable for at least 3 months.[19]

At least four further samples collected from each subject and from different areas of the synovial tissue, were immediately snap-frozen in liquid nitrogen, cryoprotected with OCT (Miles, Noperville, IL, USA) and stored at – 90°C until used for immunohistopathological studies. Another series of synovial tissue samples was used on the same day for cell cultures. The synovial tissue was dissected away from fatty, capsular components, cut into 3–5 mm pieces, washed in Hanks' balanced salt solution and incubated in collagenase (1 mg/ml) for 3 h at 37°C with constant agitation. The digest was then passed through a wire mesh with a pore size of 200 μm to separate dissociated cells from tissue debris. The cells were washed twice in collagenase and counted. The mean cell yield was $4.9 \times 10^6$ (range $0.53–16.8 \times 10^6$) and the mean viability was 88% (range: 69–98%). The cells were then cultured with RPMI supplemented with 10% FCS at 37°C in humidified 5% $CO_2$ in air. After 3 h nonadherent cells were removed and the adherent cells were cultured with RPMI supplemented with 10% FCS at 37°C in humidified 5% $CO_2$ in air for 72 h. The adherent population was subsequently stained for macrophage-specific antigens (see immunostaining section) and was found to be >80% mϕs. Every 24 h cytocentrifuge preparations ($800 \times g$ for 3 min) of suspended cells (gentle scraping) were obtained and stored at – 80°C until immunostaining.

*Immunostaining of synovial cryosections and cultured synovial macrophages:* Five μm thick cryostatic sections (Cryocut 1800, Reichter–Jung, Leika, Nussloch bei Heidelberg, Germany) of RA and control synovia as well as cytocentrifuge preparations of cultured adherent synoviocytes, were mounted on glass slides, air dried, and fixed in cold 95% acetone for 10 min. A number of slides were incubated for 1 h with a rat anti-androgen receptor mAb (IgG) that also recognizes the DNA-binding sequence of the receptor (1:100).[20] After two washes with phosphate buffered saline, the second step was performed using the improved biotin–streptavidin-amplified detection system. Briefly, staining of anti-androgen receptor mAb, was carried out using the second-step concentrated link antibody (biotinylated anti-Ig specific respectively for mouse and rat primary antibodies) and the concentrated enzyme label (horseradish peroxidase-conjugated streptavidin). The same slides were treated a second time, with mAbs directed towards single different macrophage-associated antigens Ber-MAC3 (1:70) and MAC387 (1:70), then with the second-step concentrated link antibody and finally with the concentrated enzyme label (alkaline phosphatase). Control slides were treated in an identical manner except that the first step was replaced by nonspecific mouse or rat antibodies. The samples were therefore examined by using a Leitz microscope equipped with an epifluorescence system.

*Steroid metabolism experiments:* Methodological approaches and procedures used to measure metabolic pathways of steroids have been previously established and optimized.[21] Adherent synoviocytes (see synoviocyte culture) were harvested by mild trypsinization, counted in a haemocytometer and plated onto 6-well tissue culture plates at a density of $0.5–1 \times 10^6$ cells/dish. After 24 h, cells were washed twice with PBS-A and the medium substituted with FCS-free, phenol red free RPMI medium, containing $10^{-8}$ M tritiated Tes as precursor. Controls were treated with FCS-free and phenol red free RPMI medium alone. Following 24 h incubation, medium was transferred to sterile plastic tubes (Costar) and stored at – 80°C until analysis; cells were washed three times using PBS-A and solubilized in 3 ml of 0.1% SDS at 37°C for 15–30 min. Aliquots (100 μl) of the cell lysates were therefore used to estimate DNA content, as described elsewhere.[22]

Steroid extraction was carried out using 1 ml of medium with 10 ml of dimethyl ether for androgens. After mixing at 4°C for 30 min, the resulting aqueous phase was freeze-dried in a Speed Vac evaporator-concentrator (Savant Instruments Inc., Farmingdale, NY, USA) and then resuspended using 970 μl of acetate buffer (0.75 M, pH 5.0) and 30 μl of Glusulase enzyme mixture (DuPont Co., Wilmington, DE). The solution was incubated at 37°C for 18 h to hydrolyse steroid conjugates (sulphates and glucuronides). The ether phase was evaporated to dryness under nitrogen and then stored at $-20°C$ until required for analysis using reverse phase (RP)-HPLC. The HPLC system consisted of a model 324 system, equipped with a model 160 UV detector set at 280 nm (Beckman Instruments Inc., Berkeley, CA, USA) and with 'on-line' Flo-One/beta (model 1C) three channel radiometric detector (Radiomatic Instruments, High Wycombe, Bucks, UK). Steroids were separated under isocratic conditions using a Spherisorb OS-11 (Aldrich Chimica, Milan, Italy) column ($250 \times 4.6$ mm i.d.) and either 40% or 45% acetonitrile in citric acid (0.05 M) as optimized mobile phases for oestrogen and androgen separation respectively, with a flow rate of 1 ml/min. Routine data integration was automatically achieved and computed in net cpm by a Flo-One/beta F1B program (Radiomatic, Tampa, FL, USA).

*IL-1β assay:* Culture supernatants of RA mϕs incubated in the presence or absence of Tes, were harvested after 24 h and assessed for IL-1β production. IL-1β concentrations in supernatants were measured by specific solid phase enzyme immunoassay (EIA) based on an oligoclonal system in which several monoclonal antibodies directed against distinct epitopes of IL-1β are used. Briefly, a microtitre plate precoated with several anti-IL-1β monoclonal antibodies was incubated, in duplicate, for 2 h at room temperature, with 200 μl of standards (undiluted) and synoviocyte culture supernatants (diluted 1:5 with a specific buffer solution) in the presence of a different series of monoclonal anti-IL-1β antibodies labelled with horseradish peroxidase. A chromogen (tetramethylbenzidine) was then added for 15 min after washing to detect the enzyme activity, and the reaction was then stopped by the addition of $H_2SO_4$. The amount of substrate turnover, which is proportional to the cytokine concentration, was determined colorimetrically by measuring the absorbance using a computerized reader (Vmax EASIA reader, Medgenix). Concentrations as low as 2 pg/ml of IL-1β are detectable with this assay. Cross-reaction with other cytokines including IL-1α, TNF and interferons have been reported to be insignificant. The intra-assay coefficient of variation was 7% for IL-1β.

*Statistical analysis:* Differences between samples were analysed by Student's *t*-test.

### Results

*Immunostaining of tissue cryosections and cultured cells:* The mϕs on synovial cryosections were double-labelled after double-immunostaining with the Ber–MAC3 mAb and anti-androgen receptor mAbs, respectively. Both a strong nuclear reaction for the hormone receptors and diffuse staining for the phagocytic cell antigens (Ber–MAC3), including the branched cytoplasmic extensions, were observed. This effect is indicated by the arrows in Fig. 1(A). The mean percentage of sex hormone receptor positive mϕs within the synovial lining was approximately $31 \pm 10\%$ in the rheumatoid synovia and $13 \pm 6\%$ in controls, by counting up to 200 cells in each area of the tissue examined. Mϕs were present in a smaller percentage in the sublining of all cryosection samples (about 65–70% less when compared with the lining).

When double-immunostaining was performed using cytocentrifuge preparations of cultured mϕs, and the same mAbs, an intense staining was confirmed for the AR (Fig. 1(B)). After staining for macrophage-specific antigens (Ber–MAC3 and MAC-387) over 90% of the cells from centrifuge preparations were found to be mϕs.

*Metabolism of sex hormones* in vitro *by short-term cultured human synovial macrophages:* Short-term primary cultures of RA mϕs were exposed to approximately physiological concentrations of Tes ($10^{-8}$ M) for 24 h; tritiated Tes was added to the cell cultures in order to evaluate the enzyme activity of these cells, in restrictive culture conditions (i.e., without foetal calf serum and in phenol red-free medium). Results of these three experiments showed an appreciable formation of dihydrotestosterone (DHT), the biologically active metabolite. Delta4-androstenedione (Δ4-Ad) formation, even at relatively low levels, was also detected (see Table 1). Experiments were carried out in triplicate; the reproducibility was acceptable, as revealed by the narrow standard deviation value, and good extraction efficiency was obtained (> 90% in all cases). Controls were negative for the presence of androgens and their metabolites in the medium.

*IL-1β production by cultured synovial macrophages:* To test whether testosterone modulates IL-1β production by cultured RA mϕs, the cells from five separate RA donors were incubated for 24 h in the presence of either medium alone (control cultures) or medium supplemented with physiological concentrations of Tes ($10^{-8}$ M) (IL-1β was assayed in the supernatants by a specific EIA).

As shown in Fig. 2, Tes reduced the IL-1β production by cultured mϕs by a mean of 28% (range 12–57%) of control cultures. Although in the different experiments the IL-1β production varied widely in control cultures, a significant difference in IL-1β supernatant concentration was observed between the Tes supplemented cultures when compared with the controls ($p = 0.028$; 95% confidence limits for differences 43.8 and 560.4). A large difference in basal IL-1β concentrations was related to the different clinical conditions of the RA donors evaluated.

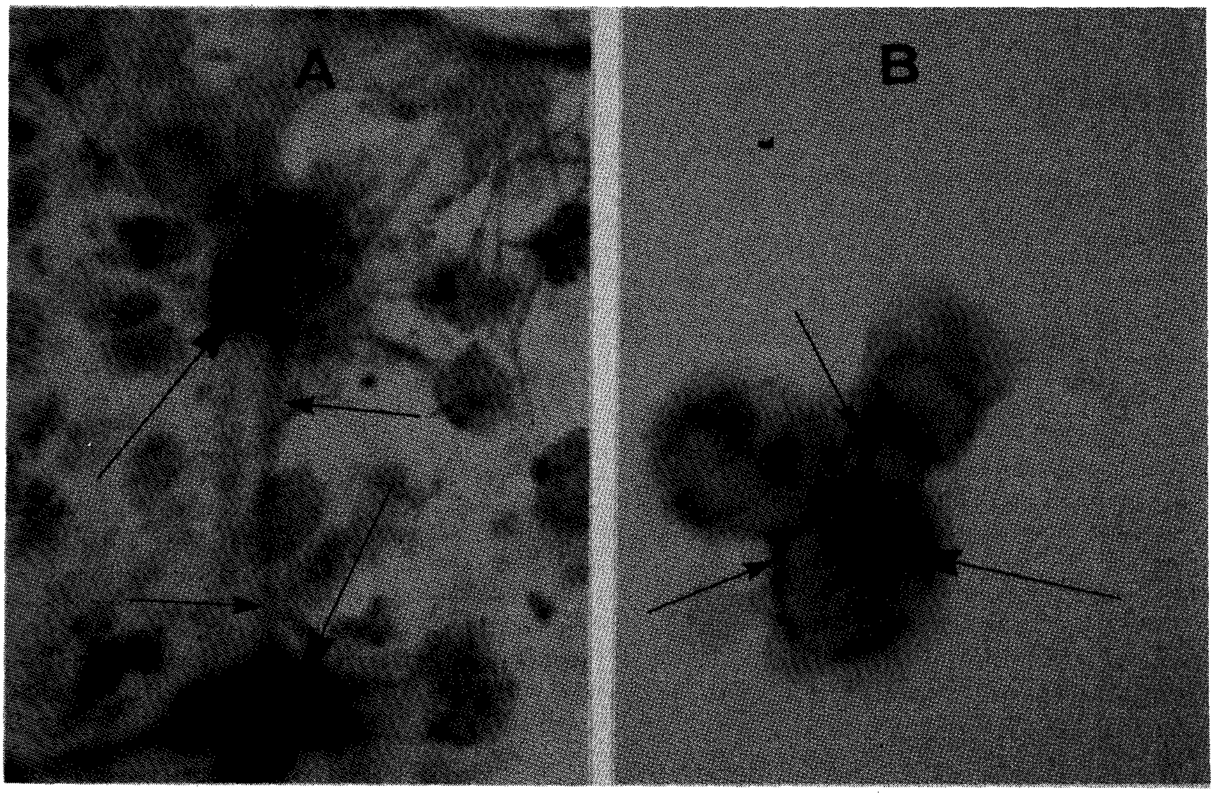

FIG. 1. Human synovial macrophages (mϕs) after double-immunostaining with the anti-AR monoclonal antibody (mAb) and the anti-phagocytic cell antigen antibody (Ber–MCA3) (cryosection, Panel A). Both a strong nuclear reaction for the AR; large arrows) and diffuse staining for the Ber–MAC3 (small arrows) are present. Cultured mϕs after double-immunostaining with the same mAbs showed a similar pattern (cytocentrifuge preparation, Panel B; original magnification ×500).

### Discussion

Previous studies have described androgen and oestrogen receptors on whole synovial tissues from healthy and RA patients and have identified the mϕs among the positive cells.[12,13] Here we show that primary cultures of mϕs isolated from human normal and rheumatoid synovial tissues of both sexes express functional androgen receptors. The immunostaining with specific mAbs of the cytocentrifuge preparations of primary cultured mϕs confirmed intense staining for AR. The cells appear also to contain the key enzymes of steroidogenesis, as shown by their capability to form, in the short term, the active metabolites of testosterone. In particular the results of the study indicate that mϕs are endowed of 5α-reductase enzymes, the latter forming DHT, the more biologically active metabolite, in relatively short-term exposure (24 h).

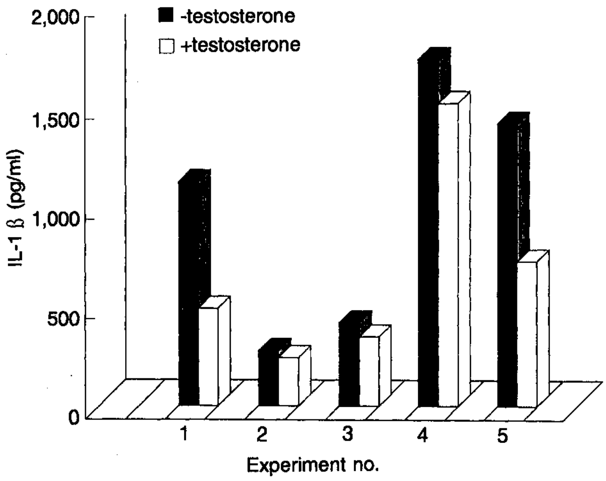

FIG. 2. Testosterone influence on IL-1β production by cultured human macrophages. Macrophages (1 × $10^{-6}$) from five separate primary cultures were cultured in 1 ml of medium without (–) or with (+) testosterone for 24 h. Supernatants have been assayed by specific solid phase enzyme immunoassay (EIA).

In cultured mϕs the rate of conversion of Tes into active metabolic products (i.e., DHT) was found very close to that observed in classical target cells for androgen activity such as human prostate cancer cells (the conversion rate was 1.4% and 1.8%, respectively).[23,24] Furthermore, DHT levels were found in physiologically relevant amounts (0.56 pmol/100 μg DNA or 7.54 × $10^{-11}$ M) as usually observed in classical target cells for peripheral androgen metabolism (i.e., human prostate cancer cells).[24]

Finally, since interleukin-1 is mainly synthesized by activated macrophages and mediates lymphocyte stimulation during immune response, it was found that testosterone treatment ($10^{-8}$ M) of cultured RA mϕs, significantly decreased IL-1β production at least at the protein level, after 24 h. Further studies on IL-

**Table 1.** Levels of free androgens, precursor and other products at 24 h in media of human cultured RA synovial macrophages

| Precursor | Tes | DHT | Δ4-Ad |
|---|---|---|---|
| [$^3$H]-Tes | *37.56 ± 0.54 (5.12 × $10^{-9}$ M) | *0.56 ± 0.02 (7.54 × $10^{-11}$ M) | *1.76 ± 0.03 (2.36 × $10^{-10}$ M) |

*Values (pmol/100 μg DNA) are expressed as mean ± S.D. of three experiments in triplicate. In brackets values expressed as molar concentrations.
Cells (1 × $10^6$) were incubated for 24 h with 1.0 × $10^{-8}$ M of [1,2,6,7-[$^3$H](N)]-Tes (specific activity, 92.1 Ci/mmol) in FCS and phenol red free RPMI medium.
(Tes = Testosterone, DHT = dihydrotestosterone, Δ4-Ad = Delta4 androstenedione).

β mRNA response are in progress. This inhibitory effect by androgens on macrophage IL-β production, was found in cultured RA mϕs obtained from patients of both sexes, therefore indicating that the effect is not sex-linked.

At the same physiological concentration ($10^{-8}$ M) employed in this assay, it is known that Tes translocates androgen receptors to cell nuclei without affecting other steroid receptors (i.e., oestrogen, progesterone) or stimulating cell growth.[25] Furthermore, recent studies, have shown that even pharmacological concentrations of Tes ($10^{-6}$ M) inhibited IL-1β secretion by peripheral blood mononuclear cells obtained from RA patients.[26]

In contrast a dose-dependent influence of oestradiol (E2) on IL-1β production by human peripheral monocytes has been described.[27] In particular, physiological concentrations of E2 ($10^{-8}$ or $10^{-9}$ M) seem to increase and pharmacological ($10^{-5}$ M) concentrations seem to inhibit IL-1β secretion. Similar results have been also obtained in primary cultures of RA mϕs (Cutolo, unpublished observations). In all these experiments, the effect of androgens on IL-1β production by monocyte/macrophages was not found to be sex-linked. In fact, physiological serum androgen concentrations, as observed in basal conditions in male subjects, may well decrease/inhibit the synthesis of IL-1β when monocyte/macrophages are activated by different stimula.

An immunosuppressive activity exerted by androgens is well assessed.[6] Therefore the lower immune response observed in males, can also explain the reduced incidence of autoimmunity such as observed in Systemic lupus erythematosus (SLE) or in RA, in which females show a much higher susceptibility.[1] In addition, as known, male patients affected by autoimmune diseases have been found to present low serum levels of androgens when compared with age-matched controls.[26]

A sex hormone relationship is well assessed with the different F:M ratios observed in various ages of women and men affected by RA (a lower RA incidence in men during fertile ages and a higher incidence in elderly men; whereas the opposite situation occurs in women).[28] Interestingly, a higher incidence of immuno-mediated diseases such as SLE, secondary Sjögren syndrome (SS) or RA is present in men with Klinefelter's syndrome more frequently than in normal men. Klinefelter's men are characterized by high oestrogen and low androgen levels.[29] Furthermore low androgens, at least in male RA patients, may be a genetically determined precursor rather than a consequence of the disease, as shown by its frequent association with the HLA antigen B15 even in healthy male controls.[30,31]

All these observations may represent the basis for the effectiveness of androgen replacement therapy in RA as well as in Klinefelter's patients.[29,32] Recently the administration of dehydroepiandrosterone (DHEA), an adrenal hormone with mild intrinsic androgenic activity, has been reported to improve the disease activity in SLE patients.[33]

In conclusion, androgens may at least partially exert their immunosuppressive activity by acting on human activated mϕs through respective receptor-mediated mechanisms.[34] These findings underscore the importance of sex hormones in immune system regulation and consequently confirm that both androgen and oestrogen imbalance may have significant implications in autoimmunity.[35–38]

### References

1. Lahita RG. Sex steroids and the rheumatic diseases. *Arthritis Rheum* 1985; **28**: 121–126.
2. Ahmed SA, Penhale WJ, Talal N. Sex hormones and autoimmune diseases. Mechanisms of sex hormone action. *Am J Pathol* 1985; **121**: 531–559.
3. Grossman C. Possible underlying mechanisms of sexual dimorphism in the immune response, fact and hypothesis. *J Steroid Biochem* 1989; **34**: 241–251.
4. Talal N. Autoimmunity an sex revisited. *Clin Immunol Immunopathol* 1989; **53**: 355–357.
5. Sthoeger ZM, Chiorazzi N, Lahita RG. Regulation of the immune response by sex hormones. *J Immunol* 1988; **141**: 91–98.
6. Schuurs AHVMM, Verheul HAM. Effects of gender and sex steroids on the immune response. *J Steroid Biochem* 1990; **35**: 157–172.
7. Harris ED. Rheumatoid arthritis: pathophysiology and implications for therapy. *N Engl J Med* 1990; **322**: 1277–1289.
8. Cohen JHM, Danel L, Cordier G, Saez S, Revillard JP. Sex steroid receptors in peripheral T cells: absence of androgen receptors and restriction of estrogen receptors to OKT8-positive cells. *J Immunol* 1983: **131**: 2767–2771.
9. Grossman CJ, Sholiton LJ, Helmsworth JA. Characteristics of the cytoplasmic and nuclear dihydrotestosterone receptor of human thymic tissue. *Steroids* 1983; **42**: 11–22.
10. McCruden B, Stimson WH. Androgen receptors in the human thymus. *Immunol Lett* 1984; **8**: 49–53.
11. Kovacs WJ, Olsen NJ. Androgen receptors in human thymocytes. *J Immunol* 1987; **139**: 490–493.
12. Cutolo M, Accardo S, Villaggio B, *et al.* Evidence for the presence of androgen receptors in the synovial tissue of rheumatoid arthritis patients and healthy controls. *Arthritis Rheum* 1992; **35**: 1007–1015.
13. Cutolo M, Accardo S, Villaggio B, *et al.* Presence of estrogen binding sites in synovial macrophages and CD8+CD29+CD45RO+ T lymphocytes in normal and rheumatoid synovia. *Arthritis Rheum* 1993; **36**: 1087–1097.
14. Arnett F, Edworthy SM, Bloch DA, *et al.* The American Rheumatism Association 1987 revised criteria for the classification of rheumatoid arthritis. *Arthritis Rheum* 1988; **31**: 315–324.
15. Lindblad S, Wredmark T. Traumatic synovitis analysed by arthroscopy and immunohistopathology. *Br J Rheumatol* 1990; **29**: 422–425.
16. Kay A, Wingrave SJ. Oral contraceptives and rheumatoid arthritis. *Lancet* 1983; **i**: 1437.
17. Tsoukas CD, Provvedini DM, Manolangas SC. 1,25 dihydroxy vitamin D3: a novel immunoregulatory hormone. *Science* 1984; **224**: 1438–1440.
18. Ettinger B, Genan HK, Cann CE. Long-term estrogen replacement prevents bone loss and fractures. *Ann Intern Med* 1985; **102**: 319–324.
19. Crawford D, Cowan S, Hyder S, McMenamin M, Smith D, Leake RE. A new storage procedure for tumor biopsies prior to estrogen receptor measurement. *Cancer Res* 1984; **46**: 2348–2352.

20. Chang C, Whelan C, Popovich J, Kokontis J, Liao S. Fusion proteins containing androgen receptor sequences and their use in production of poly- and monoclonal anti-androgen receptor antibodies. *Endocrinology* 1989; **125**: 1097–1099.
21. Castagnetta L, Granata OM, Lo Casto M, *et al.* Simple approach to measure metabolic pathways of steroids in living cells. *J Chromatogr* 1991; **572**: 25–39.
22. Leake RE, Habib F, Steroid hormone receptors: assay and characterization. In: Green R, Leake RE, eds. *Steroid Hormones: a practical approach.* Oxford: IRL Press, 1987; 67–92.
23. Carruba G, Leake RE, Rinaldi F, *et al.* Steroid growth factor interaction in human prostate cancer. 1. Short-term effects of transforming growth factors on growth of human prostate cancer cells. *Steroids* 1994; **59**: 412–420.
24. Castagnetta L, Granata OM, Polito L, *et al.* Different conversion metabolic rates of testosterone are associated to hormone-sensitive status and -response of human prostate cancer cells. *J Steroid Biochem Molec Biol* 1994; **29**: 351–357.
25. Zava DT, McGuire WL. Androgen action through estrogen receptor in a human breast cancer cell line. *Endocrinology* 1978; **103**: 624–631.
26. Li ZG, Danis VA, Brooks PM. Effect of gonadal steroids on the production of IL-1 and IL-6 by blood mononuclear cells *in vitro*. *Clin Exp Rheumatol* 1993; **11**: 157–162.
27. Polan ML, Loukides J, Nelson P, *et al.* Progesterone and estradiol modulate interleukin-1β messanger ribonucleic acid levels in cultured human peripheral monocytes. *J Clin Endocrinol Metab* 1989; **69**: 1200–1206.
28. Goemaere S, Ackerman C, Goethals C, *et al.* Onset of symptoms of rheumatoid arthritis in relation to age, sex and menopausal transition. *J Rheumatol* 1990; **17**: 1620–1622.
29. Bizzarro A, Valentini G, Di Martino G, *et al.* Influence of testosterone therapy on clinical and immunological features of autoimmune diseases associated with Klinefelter's Syndrome. *J Clin Endocrinol Metab* 1987; **64**: 32–36.
30. Cutolo M, Accardo S. Sex hormones, HLA and rheumatoid arthritis. *Clin Exp Rheumatol* 1991; **9**: 641–646.
31. Ollier W, Spector T, Silman AJ, *et al.* Are certain HLA haplotypes responsible for low testosterone in males? *Dis Markers* 1989; **7**: 139–143.
32. Cutolo M, Balleari E, Giusti M, Intra E, Accardo S. Androgen replacement therapy in male patients with rheumatoid arthritis. *Arthritis Rheum* 1991; **34**: 1–5.
33. Vollenhoven RFV, Engleman EG, McGuire JL. An open study of dehydroepiandrosterone in systemic lupus erythematosus. *Arthritis Rheum* 1994; **37**: 1305–1310.
34. Kock AE, Kunkel ST, Harlown LA, *et al.* Macrophage inflammatory protein 1α. A novel chemotactic cytokine for macrophages in rheumatoid arthritis. *J Clin Invest* 1994; **93**: 921–928.
35. Cutolo M, Balleari E, Giusti M, Monachesi M, Accardo S. Sex hormone status of male patients with rheumatoid arthritis: evidence of low serum concentrations of testosterone at baseline and after human chorionic gonadotropin stimulation. *Arthritis Rheum* 1988; **31**: 1314–1317.
36. Cutolo M, Balleari E, Giusti M, Monachesi M, Accardo S. Sex hormone status in women suffering from rheumatoid arthritis. *J Rheumatol* 1986; **13**: 1019–1023.
37. Bijlsma JW, Van Den Brink HR. Estrogen and rheumatoid arthritis. *Am J Reprod Immunol* 1992; **28**: 231–234.
38. Masi AT. Incidence of rheumatoid arthritis: do the observed age-sex interaction patterns support a role of androgenic-anabolic steroid deficiency in its pathogenesis ? *Br J Rheumatol* 1994; **33**: 697-701.

ACKNOWLEDGEMENTS. This work was supported in part by grants from the National Research Council (C.N.R.) 'Progetto Bilaterale, Italy–United Kingdom' (n° 93.00254.CT04 to M.C.), Special Project 'Ageing' (c.n. 93.435.PF40) and the Italian Ministry of the University and Scientific Research (to M.C.), AIRC (to L.C.).

**Received 25 November 1994; accepted in revised form 16 January 1995**
